# Pediatric Bone Tumors: Location and Age Distribution of 420 Cases

**DOI:** 10.3390/diagnostics14222513

**Published:** 2024-11-09

**Authors:** Sebastian Breden, Maximilian Stephan, Florian Hinterwimmer, Sarah Consalvo, Ulrich Lenze, Rüdiger von Eisenhart-Rothe, Carolin Mogler, Alexandra S. Gersing, Carolin Knebel

**Affiliations:** 1 Department of Orthopedics and Sports Orthopedics, Klinikum Rechts der Isar, Technical University of Munich, 81675 Munich, Germany; 2 German Working Group of Bone Tumors, 4031 Basel, Switzerland; 3 Institute of Pathology, School of Medicine, Technical University of Munich, 81675 Munich, Germany; 4 Department of Neuroradiology, University Hospital Munich, 81675 Munich, Germany

**Keywords:** pediatric cancer, sarcoma, musculoskeletal tumor, distribution pattern, localization, bone tumor

## Abstract

Background/Objectives: One of the most important diagnostic tools in bone tumors is X-rays. Preliminary and, in the case of some benign lesions, definitive diagnoses are formed using this basic tool. Part of the decision making in this stage is based on statistical probability using the patient’s age, as well as the incidence and predilection sites of different entities. The information used today is based on older and fragmented data. To verify the underlying principles, we retrospectively evaluated all bone tumors in children and adolescents treated by our tertiary center in the last 20 years. Methods: For this retrospective study, patients under the age of 18 years suffering from histopathologically verified bone tumors were evaluated. Data were retrieved from our local musculoskeletal tumor database. Results: We were able to include 420 children treated for bone tumors in our tertiary center. The cohort consisted of 335 benign and 85 malignant lesions. The most common lesions were 137 osteochondromas; the malignant tumors consisted mainly of osteosarcomas (53) and Ewing’s sarcomas (28). The primary predilection sites were the metaphyses of long bones. Conclusions: We were able to confirm and supplement the fragmentary data of these rare diseases using our own cohort.

## 1. Introduction

Diagnosing bone tumors is a lengthy procedure, often including many specialized tools, such as MRI, PET and biopsy-taking. However, in the beginning, there usually is a plain X-ray of the affected bone. Even though many benign tumors cannot and no malignant tumor should be diagnosed only by this first image, a preliminary diagnosis can be formed.

Differentiating bone tumors using radiological images relies heavily upon additional clinical information such as the patient’s age, the location of the lesion in the body and the exact location in the bone [1,2,3,4]. This information can be researched in the scientific literature and reviews using figures of predilection sites and ages [3,5,6]. Searching for the original sources of these known and widely used tables and images proves to be difficult. In many cases, textbooks cite older books [6] or statistically instable data [7,8]. Most of the information seems to stem from epidemiological reviews [5,7,9]. More recent publications in this field usually focus on one specific entity [10,11,12] or are reviews combining the previously mentioned entities [13,14,15,16,17].

The main reason for the lack of statistically relevant data is the rareness of primary bone tumors and the lack of systematic acquisition [18]. Although occurring more often in children than in adults [6], in 2014 only 820 new cases of malignant bone tumors were registered in the US [19]. In Germany, the incidence for malignancies around the turn of the century was reported to be around 5.5 per million for children under the age of 15 [20]. Despite the low incidence, skeletal malignancies are the sixth most common entities in cancer in children [21]. Comprehensive data about the predilection sites are sparse, especially in children. Extensive databank research resulted in only one original study summarizing all bone tumors in children around the knee treated in one specialized center [22].

As the location and age of occurrence of tumors is so important in the diagnostic work-up [2,23], we see a need for further studies confirming the existing data in use. In this retrospective study, we examine the predilection site and age of children treated at our tertiary center for bone tumors.

## 2. Materials and Methods

For this retrospective study, ethical approval was granted by the ethics committee of the School of Health and Medicine of the Technical University of Munich (N°48/20S; 21 March 2021). Written informed consent was waived due to the use of pseudonymized retrospective data.

The database of our tertiary musculoskeletal cancer center was searched for patients under the age of 18 treated for primary tumors of the bones. The German guidelines recommend referral to a specialized center as soon as there is suspicion of a tumorous condition [24,25]. This is usually performed by a general orthopedist [2].

To ensure the validity of our data, only patients with histopathologically confirmed diagnoses were included in this study. This resulted in an exclusion of all benign tumors, where radiological work-up was sufficient to find the diagnosis and no surgical treatment was needed. Cases of watchful waiting without prior biopsy, no-touch lesions and patients receiving interventional treatments were not included in this study.

For each case, the patient’s sociodemographic data, treatment history and the exact diagnosis were collected and the affected bone was discerned. The initial X-rays upon referral of each bone tumor were reviewed in two planes. For lesions in long bones, the specific location inside the bone was determined. For this study, each long bone was divided into three parts; the epi-, meta- and diaphysis. In bigger lesions, the center was determined and used to discern the exact site. The classification was conducted by two junior surgeons (S.B. and M.S.); in case of discrepancies, the decision was obtained by consensus including a senior surgeon and a senior radiologist (C.K. and A.S.G.).

Cases of multiple osteochondromas were excluded in the analyses of predilection sites. Data analysis was performed using SPSS software version 26 (SPSS, Inc., Chicago, IL, USA).

Part of the present cohort was included in a previously published study about tumors around the knee [26].

## 3. Results

Between 2003 and 2023, 518 children were referred to and underwent operative treatment at our tertiary center for tumorous lesions of the bones.

In 98 cases, a biopsy revealed no bone tumor: 82 patients suffered from tumor-like lesions and multiple osteochondromas and 2 turned out to be lymphomas. These and 15 Langerhans cell histiocytoses were excluded as hematopoietic neoplasms. The radiologically suspect lesion revealed no pathological findings upon examination eleven times. The tumor-like lesions consisted of 60 osteomyelites.

Finally, we were able to include 420 children. Resection was the main mode of therapy; chemo- and/or radiotherapy were administered where applicable. In seven cases of inoperable Ewing’s sarcoma a definitive radiotherapy was performed instead of surgical resection.

**Figure 1:**
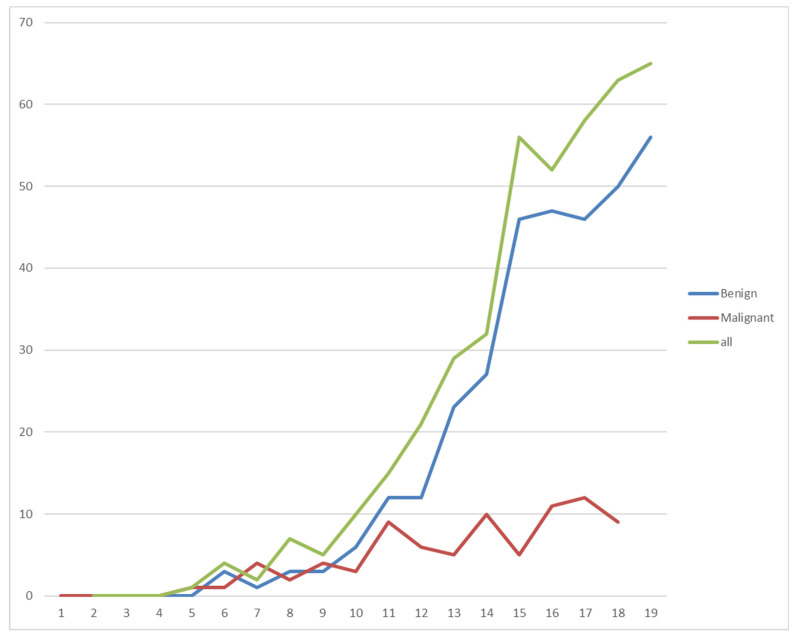
Visualization of the age distribution in our patients.

The mean patient’s age was 14.0 (3 to 18) years at the time of the surgical treatment or definitive radiotherapy. Our group consisted of 264 boys and 156 girls (ratio m/f of 1.7:1). The socio-economic data are summarized in Table 1; the age distribution is visualized in Figure 1.

In two cases soft tissue tumors (Fibrosarcoma and Fibrosarcoma) occurred in the bone; 418 children suffered from primary bone tumors, and no bone metastases of other primaries occurred. A total of 85 children (20%) presented with malignancies; 335 tumors were benign (80%).

**Figure 2:**
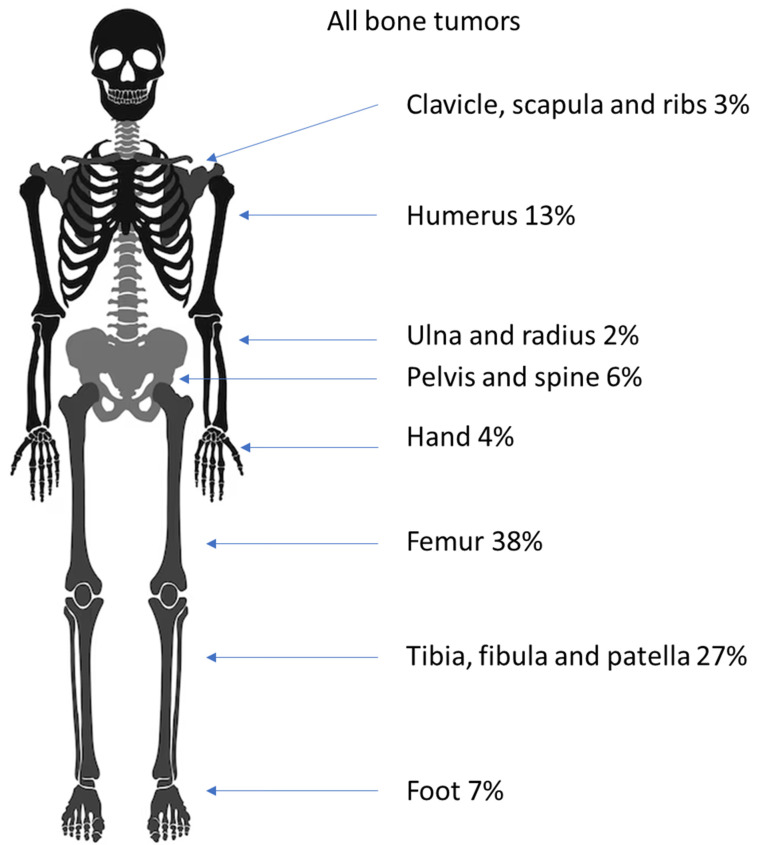
Distribution of all bone tumors in the juvenile skeleton.

**Figure 3:**
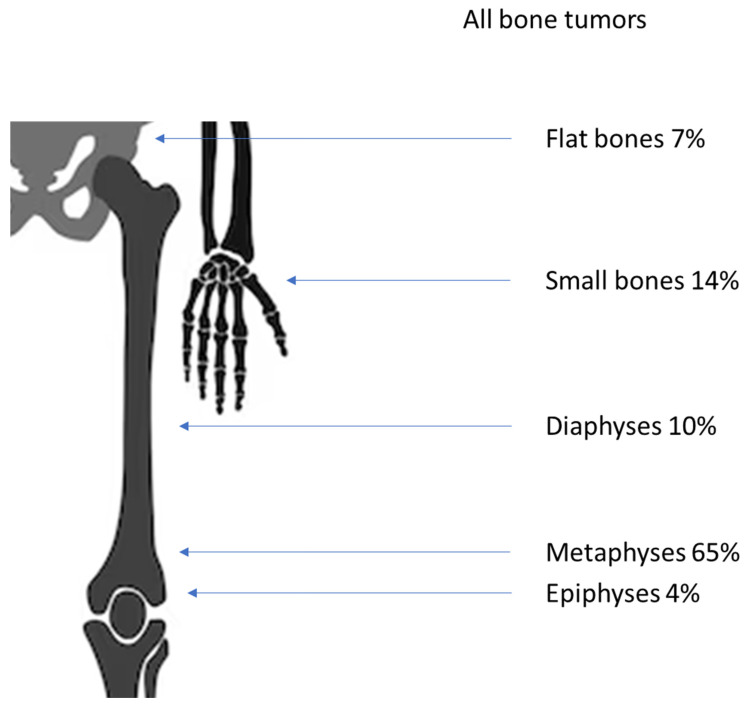
Distribution of all tumors inside the bones.

An overview of the locations of all tumors in the skeleton and in the bone is given in Figure 2 and Figure 3.

### 3.1. Benign Bone Tumors

The 335 benign tumors consisted for the most part (41%) of 137 osteochondromas. The second most common entity was 103 bone cysts (31%), which consisted of 59 simple and 44 aneurysmatic bone cysts. Other common lesions were non-ossifying fibromas (24; 7%), enchondromas (17; 5%), fibrous dysplasias (15; 4%) and chondroblastomas (13; 4%). Less often children presented with chondromas (8; 2%), of which one was sub-periostal, as well as osteoid osteomas and giant cell tumors (6; 2% each). The rarest benign tumors with under one percent each consisted of three chondomyxoid fibromas, one desmoplastic fibroma of the bone, one adamantinoma and one osteoblastoma.

**Figure 4:**
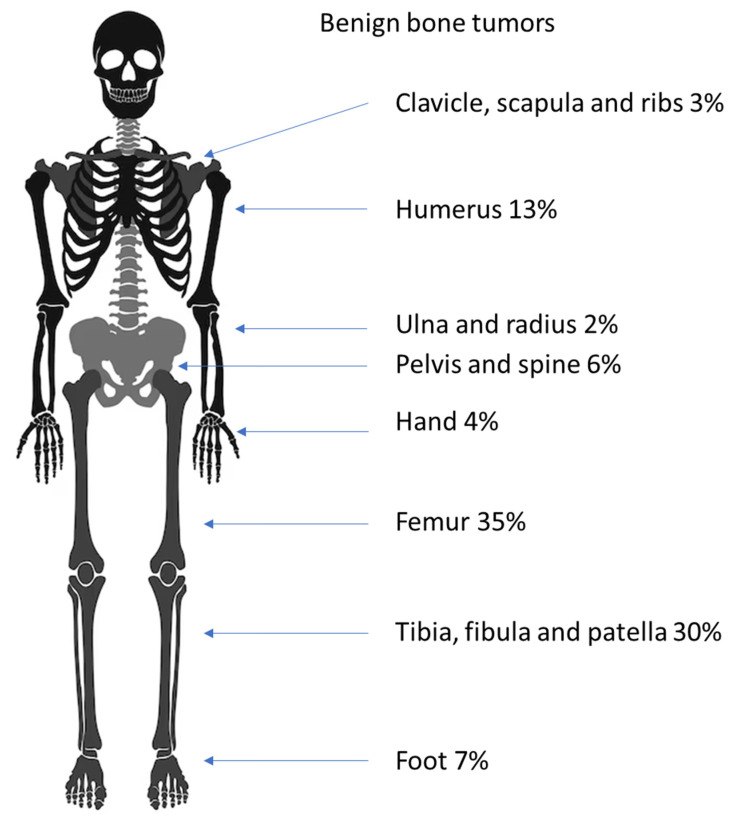
Distribution of benign bone tumors in the juvenile skeleton.

Most benign bone tumors (63%) occurred in the legs; the second most common predilection site (16%) was the arms, and tumors were found less often in the pelvis (6%), in the small bones of the feet (7%), the hands (5%) and around the shoulder (3%). For almost every entity roughly this ratio was found. The exceptions were NOFs, which were only found in the leg and the enchondromas, of which 59% were found in the hands. A detailed account is given in Table 2 and Figure 4.

The minority of the benign lesions (70; 21%) were found in small bones and the pelvis. The rest were situated in the long bones of the arms or the legs. The predilection site in the bone for most entities was the metaphyses (221; 66%); less were found in the epiphyses (14; 5%) and the diaphyses (28; 8%). There were slightly more tumors in the proximal parts of long bones than in the distal bones (133:103).

A significant amount (90%) of the osteochondromas were found in the metaphyses of long bones. The majority of simple (58%) and aneurysmal bone cysts (SBC and ABC (43%)) were detected in the metaphyses. The second predilection site was the calcaneus with 25% of all SBCs. In ABCs, the second-most number of lesions occurred in the pelvis (18%), while one was found on a rib. NOFs were only found in the long bones of the legs, with 83% in metaphyses and 17% in the diaphyses. Most of the enchondromas were located in the bones of the hand (59%); they were only secondarily located in the leg (24%). Of the fibrous dysplasias, most (47%) occurred in the femur, specifically the proximal metaphyses. Chondroblastomas were the only ones occurring mostly in the epiphyses (85%). A detailed list is given in Table 3.

**Figure 5:**
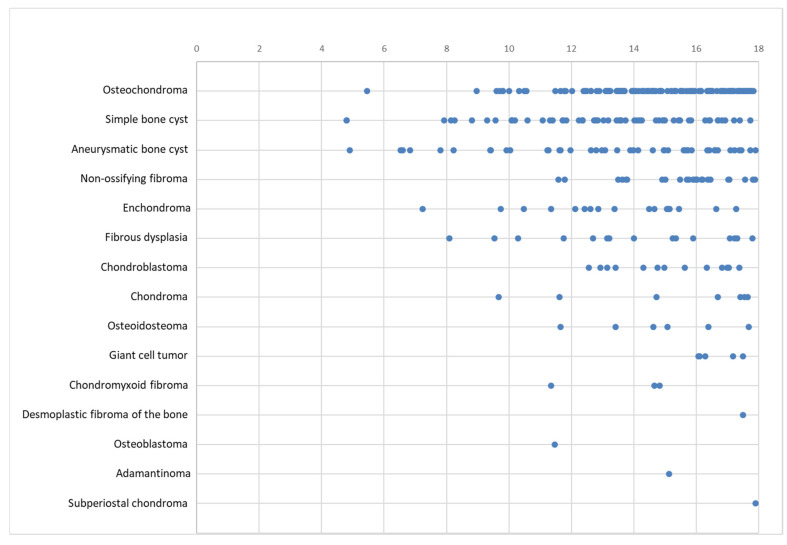
Age distribution of benign tumors in years. Each dot represents one patient.

No benign bone tumors occurred in patients under the age of four. Overall, the frequency increased with age. For osteochondromas, two peaks at 14 and 17 years can be observed, with fewer new presentations in between. Simple bone cysts were most common between 13 and 15 years of age and decreased in older children, whereas aneurysmatic bone cysts showed an almost linear increase with age. The age distribution of the benign bone tumors is shown in Figure 5.

### 3.2. Malignant Bone Tumors

The primary malignant bone tumors consisted mainly of 53 osteosarcomas (63%) and 28 Ewing’s sarcomas (33%). One atypical chondromatous tumor was found in the intertrochanteric region of a 16-year-old male, as well as one fibrosarcoma found in the proximal tibia of a 16-year-old female.

The histopathological examinations of the osteosarcomas showed mostly osteoblastic types (29; 54%). Mixed (osteo- and chondroblastic) osteosarcomas (12%) occurred less often (seven times), six were classified as telangiectatic (11%) and four were classified as chondroblastic and as high-grade (8%). The rarest subtypes were the two parosteal (4%) and one periosteal (2%) osteosarcomas.

The periosteal and one parosteal osteosarcoma were identified as grade 1 (4%). Six osteosarcomas were defined as grade 2 (11%). Most (45) were graded as grade 3 (85%).

**Figure 6:**
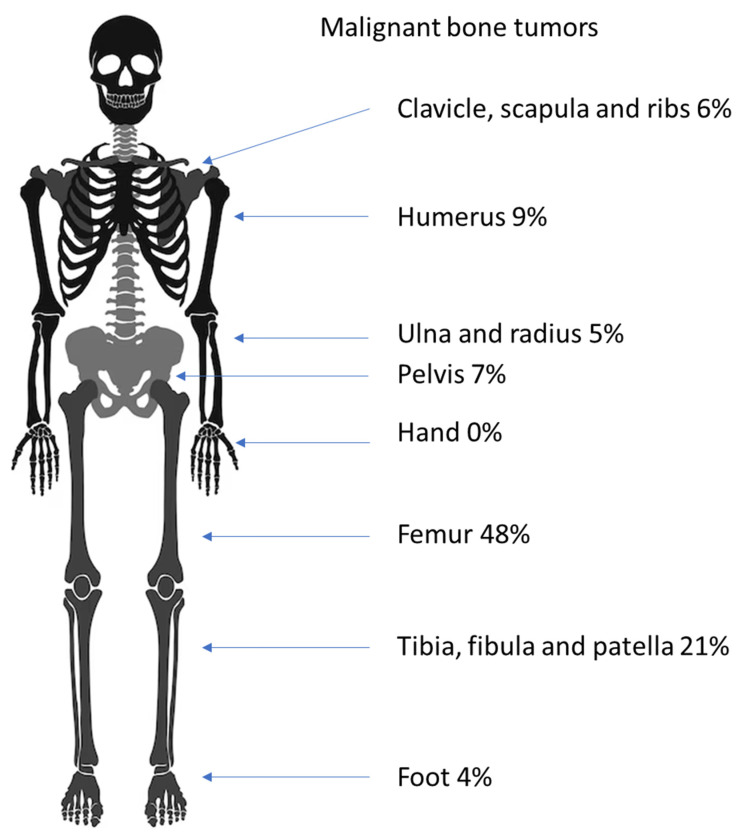
Distribution of malignant bone tumors in the juvenile skeleton.

Most of the malignant bone tumors were found in the lower extremities (63; 77%), with fewer in the upper extremities (17; 21%), and two Ewing’s sarcomas developed in the spinal column. Most osteosarcomas occurred in the long bones of the leg (42; 79%), with fewer in the long bones of the arm (9; 17%), and two occurred in the pelvis (4%). In our cohort, no other locations were affected. Most Ewing’s sarcomas were found in the long bones, with 13 in the legs (46%) and 3 in the arms (11%). Two occurred in the pelvis (7%). Other sites where Ewing’s sarcomas were found were the clavicle (five; 18%) and the calcaneus (three; 11%) (Table 4 and Figure 6).

Both types of bone sarcomas occurred most often in the metaphyses when found in long bones. A total of 38 osteosarcomas (72%) and 10 Ewing’s sarcomas (36%) were found in the metaphyses. A diaphyseal location was found in 10 osteosarcomas (19%) and six Ewing’s sarcomas (21%). Three telangiectatic osteosarcomas seemed to originate from the epiphyses (6%), but none of the Ewing’s sarcomas did (Table 5).

**Figure 7:**
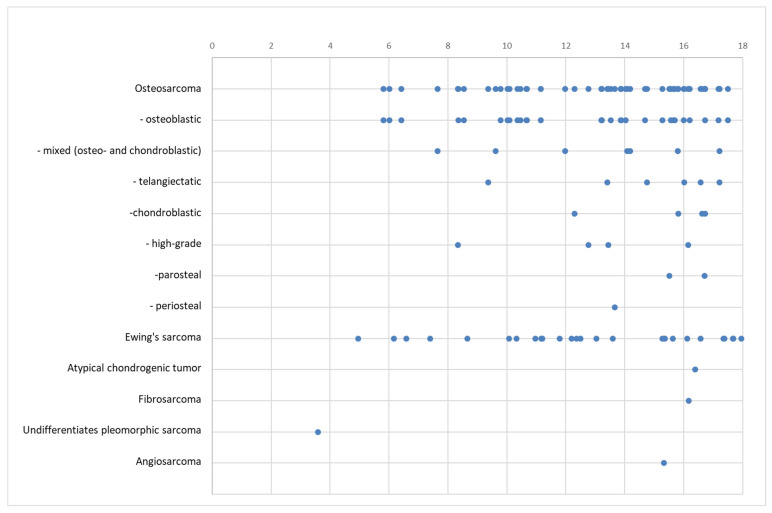
Age distribution of malignant bone tumors in years. Each dot represents one patient.

The malignant bone tumors showed a less steep increase with age in our cohort. The distribution is shown in Figure 7.

## 4. Discussion

With this study, we were able to create an updated overview of the predilection sites of bone tumors in children based on our significant dataset collected over 20 years.

As expected, the ratio of male to female patients is almost equal with a slight tendency for males suffering from primary bone tumors [7,22]. The age distribution concurs with that of previous studies, so that lesions in patients under the age of 10 are very rare and increase in frequency in the second decade of life [7,27]. The ratio of benign to malignant lesions in our cohort was more than 3:1, which was a little higher than the 2:1 ratio in Gebardt et al.’s data [22].

Nearly half (45%) of all bone tumors occurred in epi- and metaphyses around the knee. The most common predilection site in the bone was the metaphysis with almost two-thirds of all entities. This concurs with Phemister’s law, which states that the chance of bone tumors developing rises in areas with higher bone growth [28].

Tumors of the spine and the cranium are usually not treated in our center, so no reliable data on these locations are obtainable from our cohort.

### 4.1. Benign Bone Tumors

The age distribution of benign bone tumors as a whole and of individual entities in our group is similar to previously published data [5,6]. Benign tumors in children under the age of 10 are very rare (7%). The rate of occurrence rises with age, with almost one-third of all benign tumors occurring in 16 and 17 year olds (15% and 17%). No benign bone tumors were found in patients under the age of 4.

The most common entity of all primary bone tumors is, as expected, the osteochondroma [3,22]. Different from Gebhardt et al.’s study of tumors around the knee, the second most common lesion was the simple bone cyst [22]. The higher numbers in our group are probably due to the predilection site in the humerus [29]. As expected, aneurysmatic bone cysts were slightly less common than solitary ones [29]. Usually, non-ossifying fibromas are viewed as the second most common benign lesion [3,22]. In our study, the NOF ranked only fourth place as a known “leave-me-alone lesion” [30]; in most cases of NOF, no biopsy or surgical treatment was performed. These patients were not included in this study. The same has to be mentioned for osteoid osteomas, which are treated by percutaneous interventions at our center. Enchondromas are rare in children except for in the hands [31]; Gebhardt et al. reported none around the knee [22].

As expected [28], most benign bone tumors occurred in the lower extremity (77%), with 45% around the knee. The overwhelming predilection sites of osteochondromas are the metaphyses of long bones (90%), with almost half of all lesions occurring in the femur (48%). The observed predilection sites validate the published data for the formation of these tumors [28,32].

Simple bone cysts show different predilection sites. As with osteochondromas, most occur in the metaphyses of the long bones (58%), but most occur proximally (85%). The UBCs in our cohort were evenly distributed between the humerus and femur (each 24%) with predilection sites in the proximal humerus (17%), the proximal femur (19%) and the calcaneus (25%). The proximal humerus and femur are known sites for UBCs [29]. However, our consort consisted of a rather high number of calcaneal lesions, which are considered rare [29,33]. There are two possible explanations for this discrepancy. First, there could be an error in the existing data, where UBCs of the calcaneus could be misinterpreted for common calcaneal pseudocysts, which is a rarefication of trabecular bone [29,34]. Secondly, there could be a bias in our data, since, in addition to being a musculoskeletal tumor center, our institution contains a center for foot surgery.

Analogical to UBCs, aneurysmal bone cysts occurred most often in the metaphyses of the legs (41%) and the arms (16%). The rest of the ABCs were more evenly distributed than the previous entities, with 27% in small bones of the hands and feet; only one was found in the calcaneus. A large portion (18%) developed in the pelvis. A similar distribution was published by Macard et al. [29].

The non-ossifying fibromas of our cohort were all located in the long bones of the leg. NOFs in other locations are reported as rarities [27,35]. As mentioned above, it needs to be considered that most NOFs were not treated surgically and therefore do not appear in this statistic. This could especially be the case in NOFs of the upper extremity, which do not present a risk for fracture.

### 4.2. Malignant Bone Tumors

In malignant bone tumors, the age of occurrence was more evenly distributed than that in the benign cohort. The proportion of patients under ten years old was bigger than that in benign lesions (18%). The youngest patient, at 3 years, had a sarcoma. Although the rise in incidence with the patient’s age was less in the malignant cohort, most tumors occurred in older children (58% were older than 13 years).

Overall, our data on the rates of the different entities concur with previously published data [20]. The predominant primary cancers of the bones in children are osteosarcomas [27], as confirmed in our study (65%). Other authors published even higher proportions of osteosarcomas of up to 80% [22,36]. This could be due to the wider range of occurrence sites in Ewing’s sarcomas, which were not included in these studies, as we only researched tumors of the extremities. Compared to the German registry during this time, our cohort had a slightly higher rate of osteosarcomas (65% to 51%) [20].

As expected, the remaining portion of bone cancers in children consisted mainly of Ewing’s sarcomas (34%). The marginally lower rate compared to the German registry’s 46% could be due to the different age groups, with a higher incidence in the lower ages of the registry’s cohort [19]. The finding of one atypical chondrogenic tumor and one Fibrosarcoma represents a rarity [14,27].

Previously published data show the predilection site near the growth plates of long bones [7,37,38], which we were able to confirm in our cohort, where 78% were found either in the meta- or the epiphyses of long bones, mostly (79%) in the lower extremities. Ewing’s sarcomas are known to be more distributed over the body [7,8,39]. Most commonly, Ewing’s sarcomas are found in the long bones of the leg [7,8], as was the case in our cohort (46%). In our group, a sizeable portion (18%) were found in the clavicle, which is not reported that often so far [27]. Against the common rule, that Ewing’s sarcomas usually occur in the diaphyses of long bones [6,27], we saw more metaphyseal (36%) than diaphyseal (21%) lesions. The ratio of diaphyseal to metaphyseal tumors was higher in Ewing’s sarcomas than in osteosarcomas in this study (0.3:1 to 0.6:1).

### 4.3. Limitations and Outlook

Some limitations of this study need to be mentioned. One is the background of the patients that were referred to our tertiary center. This could lead to an under-representation of benign lesions, which are not referred but treated as outpatients. That limitation exists in most of the published data, since the diagnoses are too rare to be gathered from sources other than tertiary centers. In the same direction lies the fact that only histologically verified tumors were included in this study. Conducted to ensure the correctness of the diagnoses, this intensifies the under-representation of benign lesions. Another limitation in this study is the missing tumors of the spine and the cranium, which are not treated at our institution.

As in every study concerning bone tumors, the low case-count has to be mentioned as a limitation. No robust statistics can be calculated, especially for very rare entities.

It is clear that the ultimate diagnosis cannot be found solely by reviewing X-rays. Even before medical imaging, the patient’s history and symptoms have to be considered, not only for the finding of diagnoses, but for considering future treatment too [4]. In addition to the exact location in the bone, conventional radiographs allow for the analysis of bone destruction [40] and periosteal reaction [41], as well the assessment of matrix mineralization [42]. There are lesions such as non-ossifying fibromas or simple bone cysts that can be diagnosed using X-rays and medical history alone [29], but most require at least an additional MRI or even more specialized imaging [4]. Cross-sectional imaging brings further information, such as fluid levels, contrast medium enhancement and spilling into the soft tissues [4]. When a malignancy cannot be ruled out completely, a biopsy has to be taken to verify the diagnosis [33].

The diagnostic work-up for bone tumors is a very lengthy and time-consuming process that requires a multi-disciplinary workforce. Modern techniques such as AI or gene technology could hasten and facilitate the prevention, finding and treatment of bone tumors [43]. An emphasis should be on future multi-centric studies to boost patient numbers.

## 5. Conclusions

We identified the predilection site of bone tumors in children and validated previously published data. We show that the older data upon which musculoskeletal tumor surgeons and radiologists still rely are valid. Knowing the predilection sites and ages can help form a preliminary assessment of entity and dignity. Further diagnostic work-up can therefore begin earlier and the time-to-diagnosis can be shortened.

## Figures and Tables

**Table 1:** Socio-economic data of our cohort and percentages.

	Male	Female	0–4 years	5–9 years	10–14 years	15–18 years	All
All	264	156	5	39	190	186	420
	63%	37%	1%	9%	45%	45%	
Benign	219	116	3	25	155	152	335
	65%	35%	1%	8%	46%	45%	
Malignant	45	40	2	14	35	34	85
	53%	47%	2%	16%	42%	40%	

**Table 2:** Distribution of benign bone tumors.

	Hand		Foot		Clavicle + Scapula + Ribs		Humerus		Forearm		Pelvis + Spine		Femur		Lower leg + Patella		All
	Patients	Percentage	Patients	Percentage	Patients	Percentage	Patients	Percentage	Patients	Percentage	Patients	Percentage	Patients	Percentage	Patients	Percentage	
Osteochondroma	1	1%	1	1%	5	4%	21	15%	3	2%	3	2%	66	48%	37	27%	137
Simple bone cyst	0	0%	15	25%	0	0%	14	24%	0	0%	4	7%	14	24%	12	20%	59
Aneurysmatic bone cyst	1	2%	5	11%	5	11%	4	9%	3	7%	8	18%	5	11%	13	31%	44
Non-ossifying fibroma	0	0%	0	0%	0	0%	0	0%	0	0%	0	0%	5	21%	19	79%	24
Enchondroma	10	59%	1	6%	0	0%	1	6%	0	0%	1	6%	4	23%	0	0%	17
Fibrous dysplasia	0	0%	0	0%	0	0%	1	7%	1	7%	2	13%	7	47%	4	26%	15
Chondroblastoma	0	0%	0	0%	0	0%	2	15%	0	0%	1	8%	4	31%	6	46%	13
Chondroma	1	14%	0	0%	0	0%	2	29%	0	0%	0	0%	4	57%	0	0%	7
Osteoidosteoma	0	0%	2	33%	0	0%	0	0%	1	17%	0	0%	2	33%	1	17%	6
Giant cell tumor	2	33%	1	17%	0	0%	0	0%	0	0%	0	0%	2	33%	1	17%	6
Chondromyxoid fibroma	0	0%	0	0%	0	0%	0	0%	0	0%	0	0%	1	33%	2	67%	3
Desmoplastic fibroma of the bone	0	0%	0	0%	0	0%	0	0%	0	0%	0	0%	1	100%	0	0%	1
Osteoblastoma	0	0%	0	0%	0	0%	0	0%	0	0%	1	100%	0	0%	0	0%	1
Adamantinoma	0	0%	0	0%	0	0%	0	0%	0	0%	0	0%	0	0%	1	100%	1
Subperiostal chondroma	0	0%	0	0%	0	0%	0	0%	0	0%	0	0%	1	100%	0	0%	1
All benign bone tumors	15	4%	25	7%	10	3%	45	13%	8	2%	20	6%	116	35%	96	30%	335

**Table 3:** Distribution of benign bone tumors in the bones.

	Small Bones		Flat Bones		Epiphyses		Metaphyses		Diaphyses		All
	Patients	Percentage	Patients	Percentage	Patients	Percentage	Patients	Percentage	Patients	Percentage	
Osteochondroma	3	2%	7	5%	0	0%	123	90%	4	3%	137
Simple bone cyst	15	25%	4	7%	0	0%	34	58%	6	10%	59
Aneurysmatic bone cyst	12	27%	9	20%	0	0%	19	43%	4	10%	44
Non-ossifying fibroma	0	0%	0	0%	0	0%	20	83%	4	17%	24
Enchondroma	11	64%	1	6%	1	6%	3	18%	1	6%	17
Fibrous dysplasia	1	7%	1	7%	0	0%	9	59%	4	27%	15
Chondroblastoma	0	0%	1	8%	11	84%	1	8%	0	0%	13
Chondroma	1	14%	0	0%	0	0%	6	86%	0	0%	7
Osteoidosteoma	2	33%	0	0%	0	0%	0	0%	4	67%	6
Giant cell tumor	3	50%	0	0%	2	33%	1	17%	0	0%	6
Chondromyxoid fibroma	0	0%	0	0%	0	0%	3	100%	0	0%	3
Desmoplastic fibroma of the bone	0	0%	0	0%	0	0%	1	100%	0	0%	1
Osteoblastoma	0	0%	1	100%	0	0%	0	0%	0	0%	1
Adamantinoma	0	0%	0	0%	0	0%	0	0%	1	100%	1
Subperiostal chondroma	0	0%	0	0%	0	0%	1	100%	0	0%	1
All benign bone tumors	48	14%	24	7%	14	5%	221	66%	28	8%	335

**Table 4:** Distribution of malignant bone tumors.

	Hand		Foot		Clavicle + Scapula + Ribs		Humerus		Forearm		Pelvis + Spine		Femur		Lower leg + Patella		All
	Patients	Percentage	Patients	Percentage	Patients	Percentage	Patients	Percentage	Patients	Percentage	Patients	Percentage	Patients	Percentage	Patients	Percentage	
Osteosarcoma	0	0%	0	0%	0	0%	6	11%	3	6%	2	4%	30	56%	12	23%	53
- osteoblastic	0	0%	0	0%	0	0%	4	14%	2	7%	1	3%	15	52%	7	24%	29
- mixed (osteo- and chondroblastic)	0	0%	0	0%	0	0%	0	0%	1	13%	1	13%	4	49%	2	25%	8
- telangiectatic	0	0%	0	0%	0	0%	1	17%	0	0%	0	0%	4	66%	1	17%	6
- chondroblastic	0	0%	0	0%	0	0%	0	0%	0	0%	0	0%	2	67%	1	33%	3
- high-grade	0	0%	0	0%	0	0%	0	0%	0	0%	0	0%	3	75%	1	25%	4
- parosteal	0	0%	0	0%	0	0%	1	50%	0	0%	0	0%	1	50%	0	0%	2
- periosteal	0	0%	0	0%	0	0%	0	0%	0	0%	0	0%	1	100%	0	0%	1
Ewing's sarcoma	0	0%	3	11%	5	18%	2	7%	1	4%	4	14%	10	35%	3	11%	28
Atypical chondrogenic tumor	0	0%	0	0%	0	0%	0	0%	0	0%	0	0%	1	100%	0	0%	1
Fibrosarcoma	0	0%	0	0%	0	0%	0	0%	0	0%	0	0%	0	0%	1	100%	1
Undifferentiates pleomorphic sarcoma	0	0%	0	0%	0	0%	0	0%	0	0%	0	0%	0	0%	1	100%	1
Angiosarcoma	0	0%	0	0%	0	0%	0	0%	0	0%	0	0%	0	0%	1	100%	1
All malignant bone tumors	0	0%	3	4%	5	6%	8	9%	4	5%	6	7%	41	48%	18	21%	85

**Table 5:** Distribution of malignant bone tumors in the bones.

	Small Bones		Flat Bones		Epiphyses		Metaphyses		Diaphyses		All
	Patients	Percentage	Patients	Percentage	Patients	Percentage	Patients	Percentage	Patients	Percentage	
Osteosarcoma	0	0%	2	4%	3	6%	38	71%	10	19%	53
- osteoblastic	0	0%	1	3%	0	0%	25	87%	3	10%	29
- mixed (osteo- and chondroblastic)	0	0%	0	0%	0	0%	6	86%	1	14%	7
- telangiectatic	0	0%	0	0%	3	50%	1	17%	2	33%	6
- chondroblastic	0	0%	1	25%	0	0%	3	75%	0	0%	4
- high-grade	0	0%	0	0%	0	0%	2	50%	2	50%	4
- parosteal	0	0%	0	0%	0	0%	1	50%	1	50%	2
- periosteal	0	0%	0	0%	0	0%	0	0%	1	100%	1
Ewing's sarcoma	10	36%	2	7%	0	0%	10	36%	6	21%	28
Atypical chondrogenic tumor	0	0%	0	0%	0	0%	1	100%	0	0%	1
Fibrosarcoma	0	0%	0	0%	0	0%	1	100%	0	0%	1
Undifferentiates pleomorphic sarcoma	0	0%	0	0%	0	0%	1	100%	0	0%	1
Angiosarcoma	0	0%	0	0%	0	0%	1	100%	0	0%	1
All malignant bone tumors	10	12%	4	5%	3	4%	52	60%	16	19%	85

## Data Availability

The raw data supporting the conclusions of this article will be made available by the authors on request.
